# Purification and Immunobiochemical Characterization of a 31 kDa Cross-Reactive Allergen from *Phaseolus vulgaris* (Kidney Bean)

**DOI:** 10.1371/journal.pone.0063063

**Published:** 2013-05-09

**Authors:** Ramkrashan Kasera, Anand Bahadur Singh, Shakuntala Lavasa, Komarla Nagendra, Naveen Arora

**Affiliations:** 1 Allergy and Immunology Section, CSIR-Institute of Genomics and Integrative Biology, Delhi, India; 2 University of Pune, Ganeshkhind, Pune, India; 3 USLavasa Medical and Research Center, Chandigarh, India; 4 Bengaluru Allergy Centre, Bangalore, India; New York University, United States of America

## Abstract

**Background:**

Legumes are a rich source of proteins but are also potential elicitors of IgE-mediated food allergy. This study aimed to isolate and characterize a major allergen of *Phaseolus vulgaris* (kidney bean) and determine its allergenicity.

**Methodology:**

Kidney bean allergen was purified using Q Sepharose column (anion exchanger) and eluates with high intensity were pooled to purify protein using Superdex 75 (gel filtration) and C_18_ column (RP-HPLC). Patients with history of kidney bean allergy were skin prick tested (SPT) with crude kidney bean extract and the purified protein. Specific IgE was estimated in sera by enzyme-linked immunosorbent assay (ELISA). Characterization of purified protein and its cross-reactivity was investigated by immunobiochemical methods. Identification of purified protein was carried out by tandem mass spectrometry.

**Principal Findings:**

Purified protein appeared as a single band at 31 kDa on SDS-PAGE and showed IgE binding to 88% patients’ sera by ELISA and immunoblotting. SPT with purified protein identified 78% hypersensitive patients of kidney bean. Significant release of histamine from sensitized basophils was observed after challenge with purified protein. PAS staining suggested it to be a glycoprotein, but no change in IgE binding was observed after periodate oxidation. The 31 kDa protein remained stable for 60 min on incubation with pepsin. The purified protein had high allergenic potential since it required only 102 ng of self protein for 50% IgE inhibition. Mass spectrometric analysis identified it as Phytohemagglutinin. It also showed hemagglutination with human RBCs. Cross-reactivity was observed with peanut and black gram with IC_50_ of 185 and 228 ng respectively.

**Conclusion/Significance:**

A 31 kDa major allergen of kidney bean was purified and identified as phytohemagglutinin with cross-reactivity to peanut and black gram.

## Introduction

Legumes are rich source of proteins but are also potential elicitor of IgE-mediated food allergy. It has been well documented that legumes such as kidney bean, peanut, chick pea, black gram pigeon pea, lentil are an important source of IgE mediated hypersensitivity [1−5]. The clinical manifestations of the allergy to legumes range from oral allergy syndrome, urticaria, angioedema, rhinitis to asthma [6]. Recently, kidney bean has been identified as an emerging cause of food allergy among Indian population [2]. Kidney bean is consumed in Latin America, Africa, Middle East, Mediterranean area and India [1−7]. It contains high amounts of protein, abundant fibre and essential minerals like iron, zinc, calcium, and phosphorus [8]. Rouge and coworkers have also reported anaphylaxis to kidney bean [9]. Kidney bean has been identified as the major sensitizer among the atopic Indian population. Sensitization of about 22% (SPT positive) has been reported in suspected patients of food allergy [2].

So far, specific immunotherapy is the only approved curative treatment available, but it harbors drawbacks, such as side effects [10−11] and the use of crude allergen extracts [12]. Immunotherapy with crude extracts may result in additional sensitization to irrelevant components and can hamper in reaching the optimal maintenance dose during treatment. Moreover, not all the components present in extracts are allergenically relevant for diagnosis and therapy. To overcome this, use of purified and well-defined allergen preparations has been recommended [13]. Great efforts have been made to identify, characterize and purify food allergens from various sources. Three major allergens, Ara h 1, Ara h 2 and Ara h 3 [14−16], and four minor allergens, Ara h 4, Ara h 5, Ara h 6 and Ara h 7 [17], were identified from peanut. With the exception of profilin Ara h 5, these allergens are seed storage proteins. Ara h 8 is a homologue of Bet v 1 and was identified as a major allergen in birch-pollen-allergic patients with concomitant peanut allergy [18]. Oleosin, a family of proteins involved in the formation of oil bodies, was also identified as important peanut allergen. This protein also has cross-reactivity with soybean [19]. Ara h 9, a lipid transfer protein was identified as a major allergen in peanut among allergic patients from the Mediterranean area [20]. Lentil has been ranked as the fourth most important allergen in Spain, where the frequency of its allergy is high in pediatric population. Three important allergens from lentil (Len c 1, Len c 2 and Len c 3), two from soybean (β-conglycinin and n-conglycinin), Pha v 3 from green bean and Vig r 1 from *Vigna radiata* were identified, isolated and characterized [21−26].

The availability of purified proteins paves the way for component resolved diagnosis which increases the sensitivity of the test and allows the design of patient-tailored risk profile. Development and progress made in the field of purified allergens have allowed for the development of a new concept in allergy diagnosis, molecular diagnosis, which makes it possible to identify potential disease-eliciting molecules. Therefore, there is a need for well-defined purified food allergens for component resolved diagnosis which may decrease the need for provocation testing and also improve the specificity of allergen-specific immunotherapy [24]. Moreover, purified proteins can also be used to develop antibodies which could provide a valuable tool for sensitive detection of respective allergenic products in food [27]. However, further efforts should be taken to assess the range of cross-reactivity and to identify marker allergen for the most important food allergen sources. This data will affect the management of dietary requirement or recommendation of individual patient with food allergy.

Our earlier study had identified kidney bean as an important legume allergen with 15 IgE binding components. Kidney bean’s cross-reactivity has also been observed with other legumes. However, no allergen has been purified and characterized from kidney bean [2], [9]. Thus, kidney bean being the major sensitizer among atopic Indian population the present investigation was undertaken to isolate and characterize a major allergenic protein of kidney bean using column chromatographic techniques and standard immunobiochemical methods.

## Materials and Methods

### Ethics Statement

The present study protocol was approved by the human ethics committee of CSIR-Institute of Genomics and Integrative Biology, Delhi. Informed written consents were obtained from patients and non-allergic volunteers for participation in the study.

### Study Subjects

Allergic rhinitis and asthma patients with confirmed history of kidney bean allergy were included in the study from the two centres; Bangalore Allergy Centre, Bangalore and USLavasa Medical and Research Centre, Chandigarh, India. The diagnosis of asthma and rhinitis was ascertained following American Thoracic Society guidelines, 1991 and Allergic Rhinitis and its Impact on Asthma guidelines, 2001 [28−29]. Skin prick tests (SPT) were performed with kidney bean extract (1∶10 w/v) in 50% glycerinated solution. Histamine diphosphate (5 mg/ml) and PBS in 50% glycerine were used as positive and negative controls respectively. SPT was also performed with purified protein (1 mg/ml) in kidney bean sensitive patients i.e. patients skin test positive to crude kidney bean extract (n = 14). SPT reactions were observed after 20 min and wheal diameters were recorded. Patients showing SPT positivity to kidney bean with symptoms such as anaphylaxis, redness of mouth, urticaria, nausea, vomiting, diarrhea, abdominal cramps, running nose or breathlessness after ingestion of kidney bean were recruited for the study. Blood was collected from patients who showed the reversal of their respective symptoms after elimination of kidney bean from their diet (n = 25). Blood was also collected from healthy non-allergic individuals (controls, n = 5).

### Preparation of Extracts

The extraction of whole kidney bean antigen was carried out following the protocol as described earlier [30]. Briefly, healthy seeds of kidney bean were dried, crushed and defatted in diethyl ether at 4°C. The antigens were extracted in 1∶20 (w/v) ammonium bicarbonate buffer (50 mM, pH 8.0) with 5 mM ethylene diamine tetra acetate and 1 mM phenyl methyl sulfonyl fluoride by continuous stirring for 8 h at 4°C. The suspension was centrifuged at 12,000 rpm for 30 min at 4°C. The supernatant was dialyzed overnight with several changes of distilled water. The extracts were again centrifuged at 12,000 rpm for 30 min at 4°C and then passed through a 0.22 µm pore size nitro cellulose membrane filter, aliquotted in small vials and lyophilized. Protein was estimated in the extract using modified lowry’s method [31]. Similarly, the extracts for other legumes namely peanut, black gram and pigeon pea were also prepared for cross-reactivity studies.

### Purification of Kidney Bean Allergen

Three chromatographic steps were used to obtain purified 31 kDa protein.

### Ion Exchange Chromatography

Kidney bean extract was subjected to anion exchange chromatography using fast protein liquid chromatography. The extract (75 mg) was reconstituted in 20 mM Tris buffer (pH 7.9) and loaded onto Q Sepharose column equilibrated with the same buffer. After washing away the unbound protein, the bound proteins were eluted with a linear NaCl gradient (0−1000 mM). Fractions of each resolved peak were pooled, dialyzed, lyophilized and run on SDS-PAGE.

### Gel Filtration Chromatography

Pooled fractions containing allergenic protein (identified by immunoblot) were further loaded on Superdex™ 75 column prior equilibrated with 20 mM phosphate buffer, pH 7.0. Fifty fractions (0.5 mL) were collected and protein content was determined, concentrated, analyzed by immunoblot with hypersensitive pooled kidney bean patients’ sera. Allergenic protein fractions were pooled for further purification.

### Reverse-phase Chromatography

Pooled fractions (after gel filtration chromatography) were loaded on C-18 column and further purified using high pressure liquid chromatography (HPLC). Unbound proteins were washed out with distilled water till the absorbance at 280 nm became zero. The bound proteins were eluted with 50% acetonitrile in water containing 0.1% trifluoroacetic acid. The eluted fraction was analyzed by SDS-PAGE and immunoblotting using pooled sera of kidney bean hypersensitive patients.

### Estimation of Specific IgE

Levels of specific IgE in SPT positive patients’ sera was determined by enzyme linked immunosorbent assay (ELISA) by following the protocol described earlier [30]. Briefly, crude kidney bean extract or the purified protein was coated in carbonate buffer (1 µg/100 µl/well), overnight at 4°C in a microtitre plate (Nunc, USA). After washing with PBS-Tween 20 (0.05%), the nonspecific sites were blocked with 1% bovine serum albumin (BSA) for 2 h at 37°C. The plate was washed and incubated with diluted sera (1∶10 v/v) of kidney bean positive patients, overnight at 4°C. Pool of normal human sera was used as control. After washing antihuman IgE-horse radish peroxidase (1∶1000 v/v; Sigma Chemical Co., St Louis, MO, USA) was added for 4 h at 37°C. Colour was developed with orthophenylene diamine. The reaction was stopped by adding 2.5 M H_2_SO_4_ and the absorbance was read at 492 nm. Specific IgE value ≥3 times of control was considered as positive.

### ELISA Inhibition Assay

The allergenic potency of crude kidney bean extract and purified protein was determined by ELISA inhibition (competitive ELISA) using hypersensitive pooled patients’ sera as described earlier [2]. Briefly, the extract (1 µg/100 µl per well) or purified protein was coated in carbonate buffer overnight at 4°C in a microtitre plate. The kidney bean patients’ pooled serum (1∶10 v/v) for respective antigen was preincubated with 10, 50, 100, 1000 and 10000 ng of self protein at 4°C overnight and the mixture was then added to the microtitre plate coated with same extract. Normal human sera was used as control. The protein required for 50% inhibition of IgE binding was calculated using the formula given below.
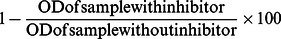



Competitive ELISA was also performed to determine cross-reactivity of purified protein with other legumes. Here, pooled patients’ sera was inhibited with different legume extracts (peanut, black gram and pigeon pea) and ELISA was performed with preinhibited sera and purified protein on solid phase.

### SDS-PAGE and Immunoblotting

The 31 kDa protein (2.5 µg) was resolved on 12% reducing gel and silver stained or commassie brilliant blue (CBB) stained for visualization.

For immunoblot, the resolved proteins were transferred on to nitrocellulose membrane as described earlier [2]. The unbound sites were blocked with 3% BSA for 3 h at 37°C. The nitrocellulose membrane strips were washed and incubated with kidney bean-hypersensitive patients’ sera (1∶10 v/v, preinhibited with bromelain) at 4°C. Normal human serum pool was taken as control. The strips were washed with PBS-Tween 20 and incubated with antihuman IgE-peroxidase (1∶1000 v/v) (Sigma Chemical Co., St Louis, MO, USA). The IgE binding was detected by diaminobenzidine with hydrogen peroxide in sodium acetate buffer (pH 5.0).

### Stripped Basophil Histamine Release Assay

Histamine release assay was performed in 15 kidney bean sensitive individuals having significantly high specific IgE values following the protocol by Kasera *et al.* [2]. In brief, peripheral blood was drawn from nonallergic donors and basophils were separated. Bound IgE was stripped off using lactic acid buffer. Subsequently, the cells were resensitized with serum IgE of individual patients (n = 15) and non allergic individuals serum as controls (n = 5). The histamine release assay was standardized using a graded amount of protein (1 ng–1 mg) and the protein concentration inducing optimal histamine release (10 ng for crude extract and 5 ng for purified protein) was determined. After passive sensitization, cells were stimulated with either kidney bean (10 ng) or with purified protein (5 ng) for 1 h. The histamine released was determined by the fluorometric method, using o-phthalaldehyde (Sigma Chemical Co., St Louis, MO, USA). Spontaneous histamine release was measured in the supernatant of unstimulated cells. The total histamine content was determined by lysis of cells with 3% perchloric acid. The allergen-induced histamine release was calculated as a percent of the total histamine content after correcting for spontaneous release. A histamine release of more than 10% was considered as positive.

### Two Dimensional Gel Electrophoresis (2-DE)

It was performed to confirm the purity of 31 kDa protein following the protocol as described earlier [32]. Purified 31 kDa protein was precipitated using 2-DE clean up kit (GE Healthcare Biosciences Corporation, Piscataway, NJ), as per instructions from the supplier. The clean protein was solubilized in rehydration buffer and applied to 7 cm nonlinear (pH 4−7) IPG strip (BioRad, CA, USA) and left overnight for rehydration. Following incubation, the strip was transferred to the focusing tray. After adding mineral oil over the strip, the separation of proteins in the first dimension was performed in an IEF cell (BioRad, CA, USA) as per manufacturer’s instructions. After focusing, a two step equilibration (reduction and alkylation) was performed. After equilibration the strip was placed over 1 mm thick 12.5% vertical acrylamide gel and held in position with molten 0.4% agarose containing bromophenol dye. The second dimension separation was performed in mini Protean III assembly (BioRad, CA, USA). After electrophoresis, the gel was subjected to silver staining.

### Silver Staining

It was performed following the method described by Blum *et al.* [33]. Briefly, the gel was incubated in fixative solution containing 50% methanol and 12% acetic acid on shaker for 1.5 h at room temperature. The fixative was removed and saved for use in later step of the experiment. The gel was washed alternatively with 50% and 30% ethanol for 30 min each. Subsequent to washing with ethanol, the gel was treated with sodium thiosulphate solution (0.002%) for 60 sec and washed thrice with distilled water for 20 sec each. The gel was placed in staining solution (12 mM AgNO_3_ and 0.028% formaldehyde) for 20 min. After incubation, the gel was washed with distilled water thrice to remove traces of silver stain and then developed. The developing solution was consisted of sodium carbonate (6%), formaldehyde (0.0185%) and sodium thiosulphate (4%). The gel was then placed in fixative solution for 10 min to stop the reaction. After that the gel was washed with double distilled water for 10 min and stored in 5% acetic acid solution. The stained gel was scanned at a resolution of 300 dpi using a scanner to acquire the image.

### Mass Spectrometric Analysis

Spots were excised from silver stained gels and digested with trypsin. The solution was injected into Agilent nanoLC-1100 (Agilent, Palo Alto, CA, USA) for analysis as described earlier [2].

### Periodic Acid Schiffs (PAS) Staining

This staining was used for detection of carbohydrate moieties in purified protein and kidney bean extract. For PAS staining, approximately 30 µg of purified protein or kidney bean extract was run on SDS-PAGE. The gel was incubated in 1% periodic acid in 3% acetic acid solution for 15 min and stained in Schiff’s reagent for 15 min in dark. After staining, the gel was washed with 5% sodium metabisulfite solution for 5 min followed by distilled water to visualize the protein bands [34].

### Periodate Oxidation

After electro-transfer of proteins onto nitrocellulose membrane strip, it was incubated in dark with 20 mM sodium metaperiodate, overnight at 4°C. Periodate was inactivated for 5−10 min by adding ethylene glycol. To this, 1 mg/ml of sodium borohydride was added and kept overnight at 4°C. The reaction was stopped by adding a drop of acetic acid. The strip was washed with water and PBS and protein free sites were blocked with 3% defatted milk in PBS. The remaining steps of western blot were carried out as described earlier [34].

### Hemagglutination Assay (HA)

Hemagglutination activity of purified 31 kDa protein was determined by HA assay. Briefly, PBS (50 µl/well) was first added to microtitre plate. To these wells, either 50 µl of purified protein (2 mg/ml) or PHA (Sigma Chemical Co., St Louis, MO, USA) was added. PBS was used as a negative control. Then 50 µl of 0.5% RBC suspension was added to each well and mixed gently. The plate was left undisturbed at room temperature for 1 h at an angle of 45°. In the absence of hemagglutination activity, the RBCs settled and appeared as dots at the corner of plates. Positive reaction resulted in a uniform reddish color across the well. HA titre was recorded as the highest dilution factor that produced a positive reading. OD of the supernatant was taken at 410 nm without disturbing the pellet.

### Heat Stability

Purified protein was boiled for 15, 30, 45 and 60 min at 100°C and analyzed by SDS-PAGE and immunoblot.

### Simulated Gastric Fluid (SGF) Digestion

The digestibility of purified kidney bean protein was examined in the SGF, as described earlier [34]. Briefly, purified protein (6.8 µg) was treated with 200 µL of prewarmed SGF (US Pharmacopoeia) containing 0.0032% (w/v) of pepsin A (Sigma Chemical Co., St Louis, MO, USA). Digestion was proceeded at 37°C with continuous shaking, and an aliquot (20 µL) of this digest was periodically withdrawn at 0.5, 1, 5, 15, 30, 45, and 60 min for analysis on SDS-PAGE.

### Immunoblot Inhibition

Immunoblot inhibition was performed to establish cross-reactivity of 31 kDa kidney bean allergen with other kidney bean proteins. Kidney bean positive sera from 7 patients’ (>1.0 O.D.) were pooled and preincubated with 100 µg of 31 kDa kidney bean protein. Whole kidney bean proteins were transferred on to nitrocellulose membrane, strips were cut, blocked and incubated with the preinhibited pooled sera. The rest of the procedure was similar to immunoblotting as described earlier in the methodology section.

### Statistical Analysis

Values are represented as mean±SD. Correlation analysis was carried out using MS EXCEL, Prism V software (Graph Pad Prism, San Diego, California, USA). Statistical significance was calculated using one way analysis of variance using software Epi Info 3.3.2. and SISA. The significance level was considered to be p<0.05.

## Results

### Purification of 31 kDa Kidney Bean Protein

**Figure 1:**
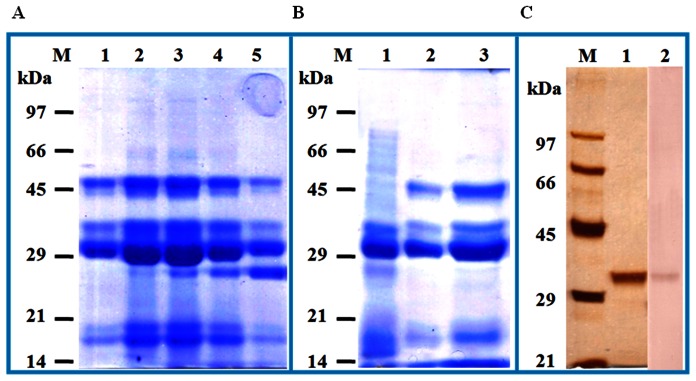
Protein profile of eluted fractions containing 31 kDa as a major eluted kidney bean protein. Kidney bean extract was subjected to anion exchange chromatography (A). Lane 1−5: fractions (10 to 14). Protein profile of gel filtration eluted fractions containing 31 kDa as a major eluted protein (B). Lane 1−3: fractions (17 to 19). Elution profile of purified 31 kDa protein after HPLC (C). Lane 1: purified protein after silver staining on SDS-PAGE, lane 2: immunoblot of purified protein using kidney bean hypersensitive pooled patients’ sera.

The adsorbed proteins of kidney bean on Q Sepharose column were eluted using NaCl gradient and fractions were run on SDS-PAGE. Fraction nos. 10 to 14 showing high intensity band at 31 kDa were pooled (Fig. 1A) and IgE binding was confirmed with immunoblotting using kidney bean hypersensitive pooled patients’ sera. The protein was further purified using gel filtration and the fraction nos. 17 to 19 with 31 kDa fractions (Fig. 1B) were pooled and purified using C_18_ columns. The major peak of interest, 31 kDa was eluted at a retention time of 4.57 min which appeared as a single band on SDS-PAGE after silver staining and was recognized by kidney bean hypersensitive pooled patients’ sera on immunoblotting (Fig. 1C).

### Allergenicity Assessment of Purified 31 kDa Protein

Specific IgE binding of purified protein (31 kDa) and crude kidney bean extract was determined by ELISA using 25 kidney bean sensitive patients’ sera (SPT positive). Specific IgE binding of 31 kDa protein, and kidney bean extract showed elevated IgE levels (OD 0.479 to 3.161) against kidney bean extract whereas 22/25 sera showed positive IgE values (OD 0.390 to 1.137) against purified protein (Table 1). There was a significant correlation of specific IgE reactivity of purified protein and kidney bean extract (r = 0.6757, p<0.01).

**Table 1:** SPT and specific IgE among the patients (1−25) and controls (C1−C5) against raw kidney bean extract and 31 kDa protein.

Patient No.	Age/Sex	Wheal area (mm^2^)			Specific IgE (O.D.)to kidney bean^a^	Specific IgE (O.D.)to 31 kDa^b^
		Histamine	Kidney bean extract	31 kDa protein		
1*	51/F	7×7	6×3	4×4	1.822	1.066
2*	34/F	5×5	6×5	n.d.	2.621	0.562
3	32/M	7×5	5×4	4×3	0.808	0.491
4*	35/M	5×6	6×5	n.d.	2.318	1.034
5	56/F	7×4	4×3	2×2	0.426	0.071
6	34/M	7×4	5×5	5×4	1.262	0.909
7	40/F	7×6	5×5	4×3	1.316	0.559
8*	52/M	6×6	6×4	3×3	2.230	0.482
9	49/M	7×5	4×3	n.d.	0.434	0.119
10	25/F	7×5	5×4	n.d.	0.839	0.577
11	60/M	7×5	5×5	n.d.	1.121	0.706
12	63/F	5×5	5×4	2×3	0.789	0.102
13	28/M	5×6	5×5	4×4	1.593	1.041
14	24/M	7×6	5×5	5×5	1.342	0.892
15	32/M	6×5	5×5	n.d.	0.919	0.622
16	48/M	6×5	4×4	n.d.	0.471	0.493
17	23/F	7×5	5×4	2×2	0.704	0.390
18	57/F	7×6	4×4	5×4	0.547	0.508
19	22/F	6×5	5×4	4×4	0.700	0.417
20	28/F	7×5	4×5	n.d.	0.565	0.533
21*	32/M	6×6	5×6	n.d.	1.631	1.046
22*	40/F	6×5	7×6	n.d.	3.161	1.137
23	48/M	5×5	3×3	3×3	0.407	0.431
24	62/F	8×4	5×4	n.d.	0.636	0.416
25	59/M	7×5	5×5	5×5	0.932	0.587
C1	28/M	5×4	1×1	1×1	0.047	0.038
C2	32/M	4×5	1×2	1×1	0.105	0.059
C3	26/F	5×4	1×1	1×1	0.087	0.052
C4	22/F	4×3	2×1	2×1	0.029	0.027
C5	38/M	3×3	1×1	1×1	0.047	0.035

a specific IgE cut off 0.189 OD.

b specific IgE cut off 0.126 OD.

* used for making patient’s pooled sera.

n.d. = SPT not done.

**Figure 2:**
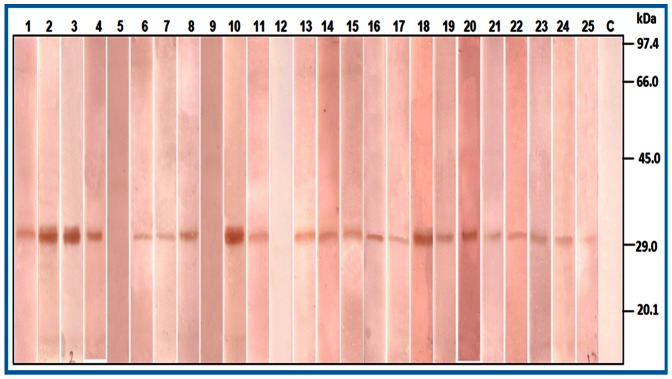
IgE binding of purified 31 kDa protein by immunoblotting. Lane 1−25: with individual patients’ sera and lane C: purified protein probed with normal human sera (control).

IgE binding of 31 kDa protein was further assessed by immunoblotting with individual patients’ sera (n = 25) having elevated specific IgE levels to kidney bean. Of these, 22 (88%) sera showed IgE binding with purified protein demonstrating it to be a major allergen (Fig. 2). Sera of three patients’ (patient nos. 5, 9 and 12) did not show IgE binding to 31 kDa protein. These patients might be sensitized to other proteins present in kidney bean.

### Biopotency of Purified Protein

Out of 25 kidney bean positive patients, 31 kDa protein was skin prick tested on 14 subjects and 11 showed marked positive skin reaction. There was a significant correlation between skin reactivity of purified protein and kidney bean extract (r = 0.615, p = 0.05). Specific IgE levels were significantly elevated in sera of all 11 SPT positive patients against both kidney bean and purified protein in ELISA (Table 1). Healthy subjects did not demonstrate positive skin reaction with any of the allergen extract.

### Histamine Release

**Figure 3:**
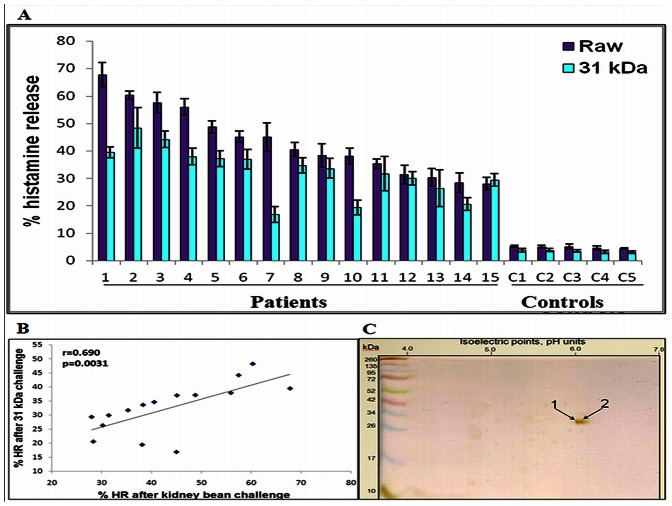
Histamine released from stripped basophils re-sensitized with individual patients’ sera (n = 15) on challenge with kidney bean extract and 31 kDa purified protein, separately. Lane C1−C5: controls (A). Scatter plot of the correlation analysis between % histamine released by patients (n = 15) and controls on challenge with crude kidney bean extract and purified protein (B). 2-DE of purified 31 kDa protein showing two close spots (1 & 2) at the same molecular weight. Both the spots were identified as PHA-E after mass spectrometric analysis (C).

Basophils sensitized with kidney bean sensitive individual patients’ sera exhibited elevated histamine release after challenge with kidney bean extract and the purified 31 kDa protein in the range of 28−67% and 17−48% respectively (Fig. 3A). Basophils sensitized with sera from healthy individuals released less than 5% histamine. Statistically significant correlation (r = 0.6903, p<0.01) was observed between histamine released by kidney bean extract and 31 kDa protein (Fig. 3B).

### Mass Spectrometric Analysis

The 31 kDa protein resolved as two discrete spots by 2-DE (Fig. 3C). Both the spots on mass spectrometric analysis were identified as erythroagglutinating Phytohemagglutinin (PHA-E). Sequence coverage of both the isoforms was more than 28%. A total of 39 peptides were matched to the database and 21 and 19 peptides showed extensive homology (p<0.05) in the respective isoforms. Phytohemagglutinin is a defensin protein which functions as host defense protein (Table 2).

**Table 2:** Peptide mass fingerprint database search of selected spots from 2-DE of purified 31 kDa kidney bean proteins.

Spot No.	Accession	Identification	Matches ofpeptides	Sequence Coverage (%)	MASCOT score	Mass/pI	Proposed function
1	P05088	Full = Erythroagglutinating phytohemagglutinin; AltName: Full = PHA-E	39(21)	32	593	29.7/5.15	defensive protein
2	P05088	Full = Erythroagglutinating phytohemagglutinin; AltName: Full = PHA-E	39(19)	28	494	29.7/5.15	defensive protein

### PAS Staining and Periodate Treatment

**Figure 4:**
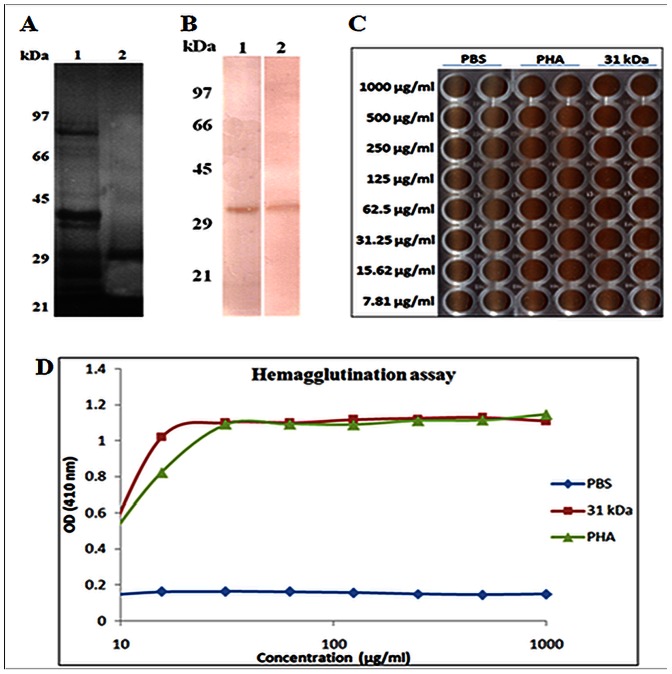
Glycoprotein detection by Periodic Acid Schiff’s (PAS) staining. Lane 1: kidney bean extract, lane 2: purified 31 kDa protein (A). Immunoblot of purified protein after periodate treatment. The 31 kDa protein was electro-transfered onto nitrocellulose membrane and then periodate oxidation was done. The strip was washed, blocked with 3% defatted milk and immunoblotted with pooled patients’ sera. Lane 1: 31 kDa protein (untreated), lane 2: 31 kDa protein after periodate treatment (B). Hemagglutination assay of 31 kDa protein, PHA (positive control) and PBS (negative control). Both 31 kDa and PHA formed uniform reddish color across the well with a minimum concentration of 15.62 µg/ml (C). Agglutination of human erythrocytes using purified protein and PHA (Sigma) (D).

The 31 kDa protein was identified as a glycoprotein after PAS staining (Figure 4A). No change in the IgE binding was observed after periodate treatment on immunoblot with patients’ sera (Figure 4B). This ruled out the possibility of any carbohydrate specific IgE binding (non specific) to 31 kDa protein.

### Hemagglutination Assay

Both 31 kDa protein and PHA (Sigma Chemical Co., St Louis, MO, USA) showed positive HA assay as compared to the negative control. Human erythrocytes remained suspended in the wells giving uniform reddish color across the well due to agglutinating activity in 31 kDa protein as well purified PHA. However, in case of PBS, erythrocytes were settled down at the corner of the plate showing absence of agglutination (Fig. 4C). The HA titre of 31 kDa was found to be 15.62 µg/ml (Fig. 4D).

### Heat Stability

**Figure 5:**
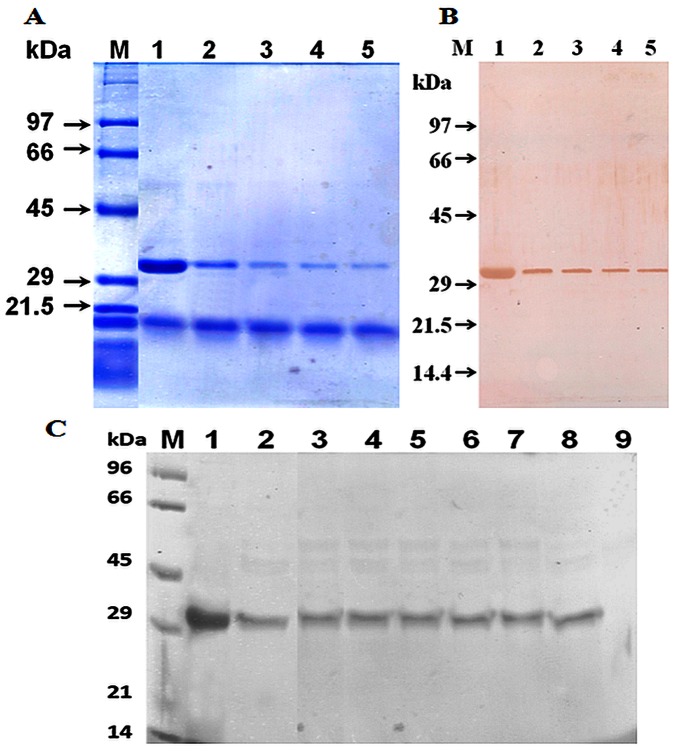
SDS-PAGE profile and immunoblot of 31 kDa kidney bean protein. Lane 1 : untreated, Lane 2−5 : boiled at 100°C for 15, 30, 45 and 60 min, Lane M: molecular weight markers. Protein fractions were stained with Coomassie brilliant blue (A) and immunoblotted (B). SGF digestion of 31 kDa protein Lane M:molecular weight markers, Lane 1: 31 kDa protein (undigested), lanes 2−8: 31 kDa protein incubated in SGF for 0.5, 1, 5, 10, 15, 30 and 60 min, lane 9: pepsin. The digested protein was electrophoresed on SDS-PAGE and visualized by CBB staining (C).

Purified protein remained stable on boiling for up to 60 min at 100°C. However the intensity of protein band decreased as the time of boiling was increased (Fig. 5A). On immunoblot purified protein showed IgE binding even after 60 min of boiling (Fig. 5B).

### SGF Digestion

Enzymatic stability of purified 31 kDa kidney bean protein was assessed by SGF digestion. The 31 kDa protein remained undigested (stable) even after 1 h treatment in SGF (Fig. 5C). The negative control, BSA was digested completely by SGF within 1 min and positive control, lactoglobulin remained stable to digestion even after 1 h of treatment.

### Allergenic Potency of 31 kDa Protein and its Cross-reactivity

The 31 kDa protein showed a dose dependent inhibition of IgE binding with self protein in competitive ELISA. A maximum inhibition of 98% was achieved with 1 µg of 31 kDa protein as inhibitor whereas 50% inhibition of IgE binding was obtained with 102 ng of self protein (31 kDa) as inhibitor.

**Figure 6:**
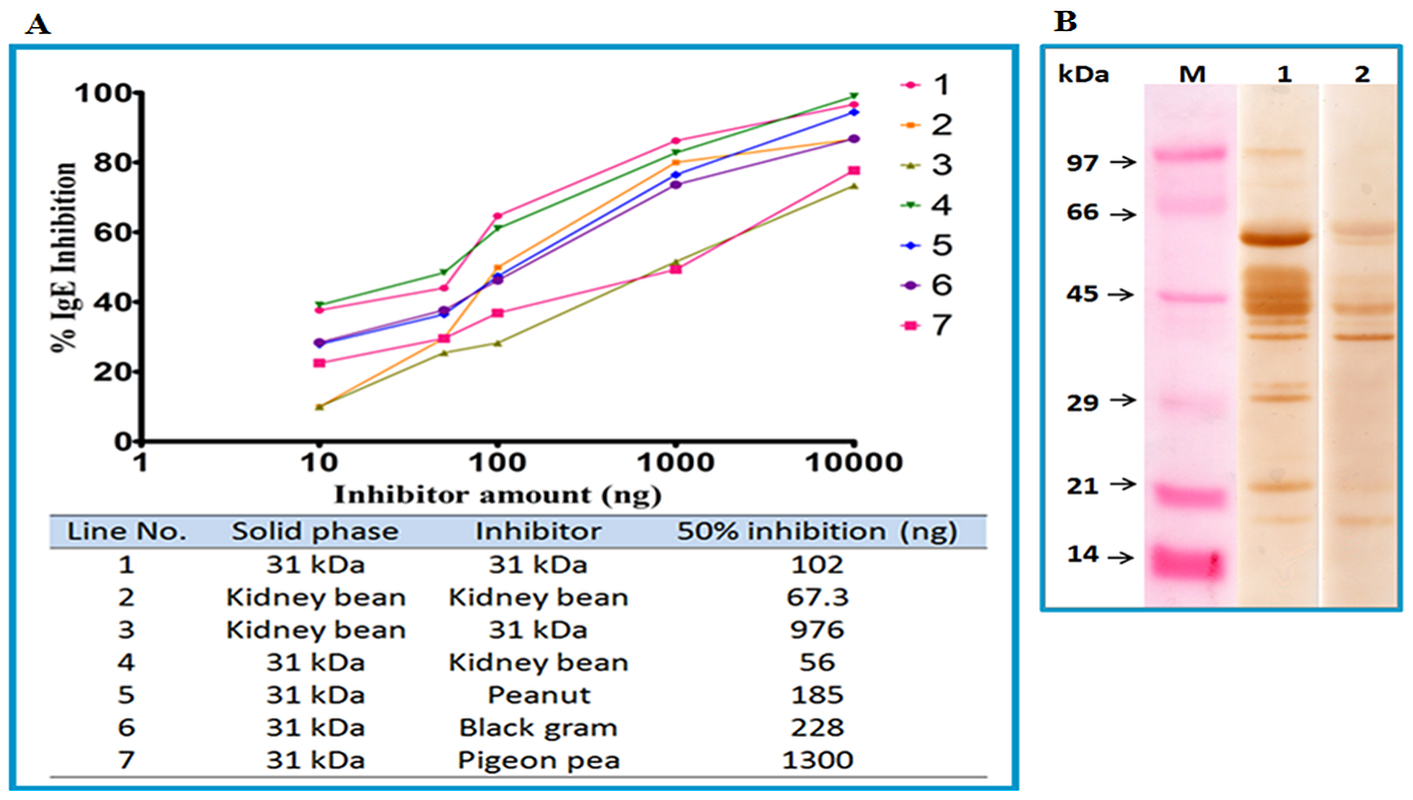
IgE ELISA inhibition of kidney bean extract and 31 kDa protein. Kidney bean protein and extracts of different legumes were used as inhibitor (A). Kidney bean positive patients’ pooled sera (1∶10 v/v) was preincubated with 5, 10, 50, 100, 1000, 10000 ng of inhibitor. ELISA was carried out on solid phase coated kidney bean extract (2 µg/100 µl/well) or 31 kDa protein (500 ng/100 µl/well) and preincubated sera. Immunoblot inhibition of kidney bean extract with 31 kDa protein (B). Kidney bean positive pooled patients’ sera was preincubated with 100 µg of 31 kDa protein (lane 2). Immunoblot using kidney bean positive pooled patients’ sera without inhibitor was used as a control (lane 1).

However, a maximum inhibition of 61% was observed in IgE binding to crude kidney bean extract (solid phase) when when 1 µg of 31 kDa protein was used as an inhibitor. Whereas, for 50% IgE inhibition to crude kidney bean extract 976 ng of purified protein was required as inhibitor. For 50% IgE inhibition to solid phase crude kidney bean extract only 67.3 ng of self protein extract was required (Fig. 6A).

Cross-reactivity of 31 kDa protein was assessed with black gram, peanut and pigeon pea by ELISA inhibition using serum pools from patients SPT positive only to kidney bean extract and negative to the tested cross-reactive antigen. Here, purified 31 kDa protein from kidney bean was coated on solid phase and different legume extracts (kidney bean, black gram, peanut and pigeon pea) were used as inhibitors. Fifty six ng of kidney bean protein (inhibitor) was required for 50% inhibition of IgE binding to solid phase 31 kDa in ELISA, whereas peanut, black gram and pigeon pea caused same inhibition with 185, 228 and 1300 ng of protein(s), respectively (Fig. 6A).

Electrophoretically transferred kidney bean proteins were incubated with preinhibited serum pool. Preabsorption of pooled patients’ sera with 100 µg of 31 kDa protein reduced its IgE reactivity (Fig. 6B, lane 2). IgE binding of proteins corresponding to 120, 95, 70, 34, 31 kDa proteins was inhibited in kidney bean indicating presence of cross reactive components. However, IgE binding of proteins separated at 55 and 50 kDa, were not completely inhibited and 45 and 42 kDa proteins remained uninhibited by purified protein. Therefore, it can be inferred that these proteins are specific allergens of kidney bean.

## Discussion

Legumes are consumed worldwide, India being at the top. In India studies on food allergens are very limited and restricted to only few foods such as egg, milk, cereals and few legumes [35−37]. Legumes such as peas, peanut, lentils, black gram, soy bean and chickpeas have been associated with severe allergic reactions [1], [18], [25], [26]. Presently, crude protein extracts are used in the allergic diagnosis. But their use has several pitfalls like batch to batch variation, low expression or concentration of many important allergens in whole extracts. Apart from this many endogenous enzymes may degrade allergens during extraction procedures. However, these problems can be overcome by using purified protein for component resolved diagnosis and patient-tailored therapy [38].

Over the years, many allergens have been purified from diverse sources such as pollen, fungi, insects, mites, and foods including legumes. These have also proved promising in selective diagnosis/therapy of allergy. For example, the major allergen Bet v 1 from birch pollen was purified from natural sources by employing ammonium sulfate precipitation, hydrophobic interaction chromatography and size exclusion chromatography [39]. Marsh *et al.* [40] purified native peanut allergens Ara h 1, 2, 3, 4 and 6 for diagnosis of peanut allergy. Ara h 1, a 63 kDa protein was purified on a ConA Sepharose-4B affinity column after ammonium sulfate precipitation. Ara h 2 and 6 were purified after ammonium sulfate precipitation and gel filtration followed by anion exchange and reverse phase HPLC. Ara h 3/4 were purified following anion exchange and gel filtration chromatography. A major allergen from soy (Gly m Bd), was purified by immunoaffinity column using mAb as a ligand [41]. Anion-exchange chromatography and reverse-phase HPLC were also used to purify Len c 1.01- a major allergen from lentil [42]. A seed storage 24 kDa protein was purified from the seeds of *Lathyrus sativus* by ammonium sulfate fractionation and ion-exchange chromatography [43]. Another 25 kDa legume protein from *Vigna unguiculata* extract was fractionated using ammonium salt precipitation followed by gel chromatographic separation which led to separation of this protein in pure form [44]. Moreover, for component resolved diagnosis with kiwifruit allergy, 4 specific marker allergens were identified out of the panel of 11 kiwifruit allergens and showed the potential of component resolved diagnosis in increasing sensitivity and specificity of *in vitro* tests [45].

In the present study, we have used combination of anion exchange and gel filtration chromatography on FPLC followed by reverse phase HPLC on C_18_ column to get purified 31 kDa kidney bean allergen. Moreover, IgE binding of the purified 31 kDa protein was assessed by *in vivo* (SPT), *in vitro* (ELISA and immunoblotting) and *ex vivo* (histamine release assay) methods. The allergenic relevance of purified 31 kDa protein was showed by IgE binding with more than 88% of kidney bean sensitive patient’s sera. Later, when a subset of 14 sensitive patients out of 25 were skin tested, 11/14 (78%) kidney bean sensitive patients showed SPT positive reaction to 31 kDa protein. Thus, being a major allergen it can be used as a marker in the diagnosis of kidney bean allergy. Purified protein inhibited up to 73% IgE binding in competitive ELISA when 10 µg of purified protein was used as inhibitor and crude kidney bean extract on solid phase. Earlier, in a case report by Schiavino et al. [46], anaphylaxis has been reported after SPT with phytohaemoagglutinin.

In the present study, 31 kDa protein demonstrated significant histamine release from kidney bean positive patients’ samples (n = 15) as compared to controls, indicating that purified protein is a potent allergen having clinical relevance in diagnosis. Specific IgE values and histamine release with purified 31 kDa protein and kidney bean extract correlated significantly (p<0.01). This suggested the importance of purified allergen and its use for diagnosis and therapy of kidney bean allergy. In an earlier study by Kumari *et al.* [34], 7/9 black gram sensitive patients were found SPT positive to its 28 kDa purified protein whereas, 77% black gram patients showed significant IgE binding. López-Torrejón *et al.* [42] in their study on lentil and its purified protein, showed IgE binding of Len c 1.01 to ≥80% of the patient’s sera.

In the present study, 102 ng of 31 kDa protein was required for 50% inhibition of self protein whereas, 976 ng of 31 kDa protein was required for kidney bean extract showing it to be a potent allergen. Cross-reactivity has been reported previously among legume allergens [1], [2], [47]. In the present study, 56 ng of kidney bean extract was required to obtain 50.0% inhibition of IgE binding on solid phase 31 kDa, whereas 185 and 228 ng of peanut and black gram were required respectively for the same. In another study inhibition of 65% IgE binding was observed with 2 µg of purified lentil protein as inhibitor and whole lentil extract on solid phase in immunoblot inhibition [42]. IgE binding protein components of kidney bean corresponding to 120, 95, 70, 34 kDa were completely inhibited when pooled patients’ sera was preabsorbed with 100 µg of self protein in immunoblot inhibition. Inhibition of these proteins suggests presence of shared epitopes.

Resistance to proteolytic enzymes and heat is a property associated with food allergens. Many food allergens or stable allergen fragments have been shown to resist conditions of the gastrointestinal tract, and thus have the potential to sensitize the immune system. In the present study, pepsin digestion of 31 kDa protein showed no cleavage products in the SDS-PAGE. This suggested that the protein is available to be taken up by the immune cells and have role in sensitizing system. However, Kumari *et al.* [34] showed the formation of stable protein fragments of 14−16 kDa after digestion with pepsin. The 31 kDa protein was also found stable to heat as the protein is visible on SDS-PAGE even after 1 h of boiling and also showed IgE binding on immunoblot. This further provides evidence that heat stable proteins are much more likely to be allergenic. Rouge *et al.* [9] identified phaseolin and PHA as putative allergens in kidney bean. Both phaseolin (>150 kDa) and PHA (120 kDa) were resistant to heat denaturation and digestive proteolysis, exhibiting an extended surface susceptible to display IgE-binding epitopes that probably account for their allergenic propensity.

The purified 31 kDa protein was identified as PHA-E after mass spectrometric analysis which is a defensive protein. PAS staining identified it as a glycoprotein. Periodate oxidation did not reduce the IgE binding of 31 kDa protein as observed on immunoblot. Thus, ruling out the possibility of any non-specific binding due to the presence of carbohydrate moieties. The 31 kDa protein in the present study showed agglutination activity similar to commercial PHA obtained from Sigma.

In conclusion, a 31 kDa kidney bean protein was purified and identified as phytohemagglutinin. This may be implicated in possible strategies for component resolved diagnosis and therapy of kidney bean allergy. However, further *in vivo* studies are warranted with purified 31 kDa protein to confirm its contribution to various symptoms of food allergy.
